# Early Life Stress Predicts Depressive Symptoms in Adolescents During the COVID-19 Pandemic: The Mediating Role of Perceived Stress

**DOI:** 10.3389/fpsyg.2020.603748

**Published:** 2021-01-12

**Authors:** Ian H. Gotlib, Lauren R. Borchers, Rajpreet Chahal, Anthony J. Gifuni, Giana I. Teresi, Tiffany C. Ho

**Affiliations:** ^1^Department of Psychology, Stanford University, Stanford, CA, United States; ^2^Department of Psychiatry, Douglas Mental Health University Institute, McGill University, Montréal, QC, Canada; ^3^Department of Psychiatry, Weill Institute for Neurosciences, University of California, San Francisco, San Francisco, CA, United States

**Keywords:** early life stress, COVID-19, adolescence, depression, perceived stress

## Abstract

**Background:**

Exposure to early life stress (ELS) is alarmingly prevalent and has been linked to the high rates of depression documented in adolescence. Researchers have theorized that ELS may increase adolescents’ vulnerability or reactivity to the effects of subsequent stressors, placing them at higher risk for developing symptoms of depression.

**Methods:**

We tested this formulation in a longitudinal study by assessing levels of stress and depression during the COVID-19 pandemic in a sample of adolescents from the San Francisco Bay Area (*N* = 109; 43 male; ages 13–20 years) who had been characterized 3–7 years earlier (*M* = 5.06, *SD* = 0.86 years) with respect to exposure to ELS and symptoms of depression.

**Results:**

As expected, severity of ELS predicted levels of depressive symptoms during the pandemic [*r*(107) = 0.26, *p* = 0.006], which were higher in females than in males [*t*(107) = −3.56, *p* < 0.001]. Importantly, the association between ELS and depression was mediated by adolescents’ reported levels of stress, even after controlling for demographic variables.

**Conclusions:**

These findings underscore the importance of monitoring the mental health of vulnerable children and adolescents during this pandemic and targeting perceived stress in high-risk youth.

## Introduction

A growing body of research is documenting the adverse consequences of exposure to early life stress (ELS) for mental health across the lifespan (McLaughlin, 2016; LeMoult et al., 2019b). In particular, ELS has been implicated in the elevated rates of depression during adolescence (LeMoult et al., 2019b). Indeed, recent epidemiological data from over 100,000 adolescents ages 12–17 years indicate an incidence of 25% for Major Depressive Disorder (MDD), with females exhibiting about twice the incidence of MDD as males (Breslau et al., 2017); further, almost half of adults with depression have their first episode during adolescence (Birmaher et al., 2002). Importantly, adolescents who experience depression are significantly more likely as adults to have more mental and physical health illnesses, lower levels of educational attainment, lower salaries, more difficulties in their relationships, and more contact with the criminal justice system (World Health Organization, 2012).

Given the alarmingly high prevalence of ELS (Child Welfare Information Gateway, 2019; Kim and Drake, 2019) and its significant impact on mental health in youth, it is critical that we elucidate mechanisms by which ELS increases risk for depression. One possibility is that exposure to ELS increases individuals’ reactivity to subsequent stressors and, thus, also the likelihood that they will experience depression (Post, 2007). Indeed, the odds of reporting psychological distress increase steeply with the number of prior adverse experiences, suggesting that earlier stressful experiences potentiate the impact of later stressful experiences (Manyema et al., 2018). This possibility is especially salient now, given the severe acute respiratory syndrome coronavirus 2 (SARS-CoV-2 or COVID-19) pandemic, which has resulted in personal and familial distress, economic hardship, and enforced social isolation (Galea et al., 2020; Holmes et al., 2020). Indeed, youth are experiencing significant disruptions in their normal daily routines, their face-to-face, peer interactions, and are likely to be particularly susceptible to the stress caused by COVID-related changes and restrictions (Gunnell et al., 2020; Orben et al., 2020). Already, and perhaps not surprisingly, early reports show that 83% of youth (younger than 25 years) with a history of mental illness believe that their conditions are worsening (Lee, 2020). It is unclear, however, whether and how the effects of the pandemic will affect adolescents who do not have significant psychiatric histories, and relatedly, whether exposure to ELS will render adolescents more vulnerable to the stress of the pandemic. Identifying groups of young individuals who experience significant distress in the face of such a stress will facilitate the efficient deployment of prevention efforts such as screening for mental health problems, and in the mobilization of supports such as psycho education and online or remote interventions for those at risk for developing depression and other mental health difficulties (Pfefferbaum and North, 2020). In this paper, we report findings from a longitudinal study in which we examined whether adolescents who experienced more severe ELS as children report higher levels of depressive symptoms during the pandemic, and whether this association is mediated by elevated levels of perceived stress during the pandemic.

## Materials and Methods

### Participants and Procedures

**FIGURE 1:**
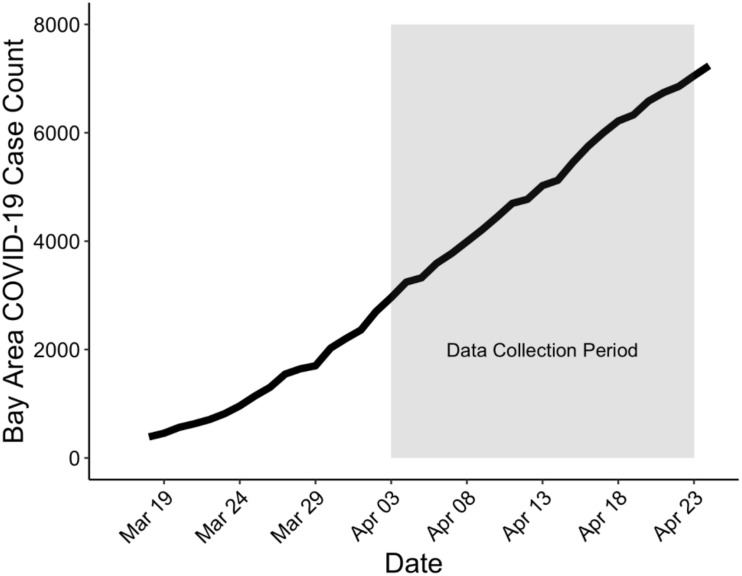
Timeline of data collection in the current study (highlighted in gray) relative to rising cases in the San Francisco Bay Area. Shelter-in-place was issued between March 16–18, 2020. The first participant completed the survey April 3rd, 2020 and the last participant completed the survey April 23rd, 2020. COVID-19 cases were taken from https://data.ca.gov/ dataset/covid-19-cases from the following counties: Alameda, Contra Costa, Marin, Napa, San Francisco, San Mateo, Santa Clara, Solano, and Sonoma.

From 2013–2016, we recruited 214 children and adolescents and their parents from the San Francisco Bay Area using media and online advertisements to participate in a longitudinal study of the psychobiological effects of ELS across the transition through puberty (King et al., 2019; LeMoult et al., 2019a; Miller et al., 2020). Inclusion criteria were that adolescents were ages 9–13 years and were proficient in spoken English. Exclusion criteria included factors that would preclude an MRI scan (e.g., metal implants, braces, and claustrophobia), any history of major neurological or medical illness, severe learning disabilities, and, for females, the onset of menses. Following recruitment, four participants were excluded from the final sample (2 withdrew, 1 had a medical illness, and 1 did not respond after initial contact), resulting in 221 participants available for the current study. At baseline, we assessed ELS and depressive symptoms in all adolescents (see section “Measures”). Between April 3 and April 23, 2020, approximately 3 weeks (*M* = 22.57 days, *SD* = 4.94 days) after shelter-in-place was implemented in the Bay Area on March 16–18, 2020, adolescents completed online questionnaires assessing depressive symptoms, perceived levels of stress, and exposure and impact of COVID-19 on their behaviors and emotional well-being (see Figure 1 for a timeline of this assessment). Of the 221 eligible participants, 109 (49.32%) completed the survey [43 males; ages 12.82–19.98 years (*M* = 16.30, *SD* = 1.47)]. In accordance with the Declaration of Helsinki, participants and their parents provided informed written assent and consent, respectively. This study was approved by the Stanford University Institutional Review Board and all participants were compensated for their participation.

### Measures

#### Baseline Assessment: Early Life Stress

In addition to obtaining other information and measures at the time of study enrollment, all participants were interviewed at baseline, 3–6.5 years prior to COVID-19 (*M* = 5.06, *SD* = 0.86), using a modified version of the Traumatic Events Screening Inventory for Children (Ribbe, 1996). Modifications are described in detail in King et al. (2017); briefly, participants were asked whether they had experienced any of over 30 different types of stressful events (e.g., “Have you ever experienced a severe illness or injury?,” “Have your parents ever separated or divorced?”). If a participant endorsed having experienced a stressful event, interviewers followed up with specific questions to contextualize the event (e.g., timing, duration, frequency, and involvement of others). For each event that a participant endorsed, a panel of three coders, blind to the participant’s reactions and behaviors during the interview, rated the objective severity of the event using a modified version of the UCLA Life Stress Interview coding system (Rudolph et al., 2000) based on a 5-point scale. To compute the severity of ELS exposure for each participant, we summed the maximum objective severity rating score for each type of stressor endorsed in order to not overweight reports of frequent but less severe events (King et al., 2017).

#### Baseline Assessment: Depression Symptoms

At baseline, participants completed the 10-item version of the Children’s Depression Inventory (CDI-S; Kovacs, 1985), a self-report measure of depressive symptoms designed for youth ages 8–16 years based on a 30-point scale (*M* = 1.77; *SD* = 2.05, reflecting very low levels of depressive symptoms at baseline). Responses capture symptoms during the past two weeks (e.g., “I am sad all the time,” “I feel alone all the time”). Studies have reported high validity and reliability for this measure (Allgaier et al., 2012). Across the full sample, internal consistency of the CDI-S was high (Cronbach’s α = 0.75).

#### COVID-19 Assessment: Depressive Symptoms, Stress, and Impact of COVID-19

To assess the impact of COVID-19 and the related quarantine (“shelter-in-place”) and isolation (social distancing) directives on health outcomes, we sent online questionnaires to all participants. Adolescents also completed the **Center for Epidemiology Studies-Depression for Children scale** (CES-DC; Weissman et al., 1980) a well-validated measure of depressive symptoms used for older children and adolescents (Faulstich et al., 1986), capturing the age range of our sample during COVID-19. The CES-DC is a 20-item measure based on a 4-point scale; responses capture symptoms experienced over the last week (e.g., “I felt down and unhappy,” “I felt like crying”). Participants also completed the **Perceived Stress Scale** (Cohen et al., 1983), a 10-item questionnaire based on a 5-point scale. The PSS is a widely used scale for assessing perception of stress in response to life events. Responses capture symptoms and perceptions experienced over the past month (e.g., “how often have you felt nervous and “stressed,” “how often have you found that you could not cope with all the things that you had to do”). In the current sample, internal consistency of the CES-DC and PSS were high (Cronbach’s α = 0.91 and 0.84, respectively). One male participant did not complete the PSS. Finally, we administered a subset of items from the youth version of the **Coronavirus Health Impact Survey** (CRISIS v. 0.2^1^), recently developed by researchers at the National Institute of Mental Health and the Child Mind Institute. The CRISIS assesses exposure to and symptoms of COVID-19, impact on behaviors, and emotions/worries. With respect to physical symptoms, we asked participants whether in the last two weeks they have experienced fever, cough, shortness of breath, sore throat, fatigue, or loss of taste or smell.

#### Statistical Analyses

**FIGURE 2:**
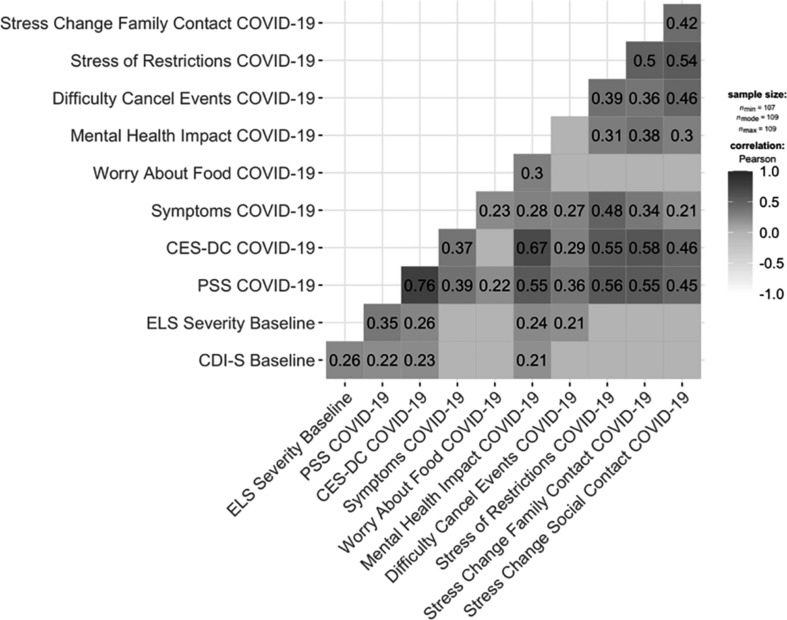
Correlations among Baseline and COVID-19 items/measures assessed in this study. CDI-S, Children’s Depression Inventory; ELS Severity, early life stress; PSS, Perceived Stress Scale; CES-DC, Center for Epidemiologic Studies Depression Scale for Children. Content of COVID-19 items: **Symptoms:** self-reported fever, cough, shortness of breath, sore throat, fatigue, loss of taste or smell; **Worry About Food:** “did you worry whether your food would run out because of a lack of money?”; **Mental Health Impact:** “During the past two weeks, how worried have you been about your mental/emotional health being influenced by Coronavirus/COVID-19?”; **Difficulty Cancel Events:** “how much has cancellation of important events (such as graduation, prom, vacation, etc.) in your life been difficult for you in the past two weeks?”; **Stress of Restrictions:** “how stressful have the restrictions on leaving home been for you?”; **Stress Change Social Contact:** “how stressful have these changes in your social contacts been for you?”; **Stress Change Family Contact:** “how stressful have these changes in family contacts been for you?”. Correlations are corrected using False Discovery Rate; only significant correlations are shown.

All data were analyzed using R (R Core Team, 2019). We first compared adolescents’ levels of ELS and CDI-S scores at baseline to test differences between adolescents who participated in the COVID-19 assessment and those who did not. We then compared male and female adolescents’ scores on the baseline and COVID-19 measures. We conducted bivariate Pearson’s correlations, corrected for multiple comparisons, to assess the strength of association among the baseline measures, CRISIS items, and the COVID-19-assessed PSS and CES-DC (Figure 2). Finally, to determine whether perceived stress accounted for the association between severity of ELS and CES-DC scores, we conducted a mediation analysis and computed 95% confidence intervals (CI) of the indirect effect of perceived stress with bootstrapping (5,000 resamplings) using the “mediate” function from the *psych* library in R (Revelle, 2017). The mediation analysis controlled for age at baseline, CDI-S scores at baseline, sex, race, parental income, and age at the COVID-19 assessment; we conducted a follow-up moderated mediation analysis to determine whether the pattern of results was the same for male and female participants. All variables were z-scored and all parametric statistical tests were two-tailed (α = 0.05).

## Results

### Demographic Characteristics and Sex Differences

Demographic characteristics of the sample by sex and statistical tests and significance levels are presented in Table 1. Male participants were older than female participants at baseline and at the COVID-19 assessment. Male and female participants did not differ in the severity of ELS at baseline; however, female participants had higher CDI-S scores at baseline. Adolescents were racially and ethnically diverse; 58% self-reported as non-white. Household yearly income ranged from <$10,000 to ≥$150,000. Female participants scored higher on the PSS and CES-DC at the COVID-19 assessment than did male participants.

**TABLE 1:** Descriptive statistics of study sample by sex.

*Total N* = 109	Male (*n* = 43) *M*(*SD*) or *N*(%)	Female (*n* = 66) *M*(*SD*) or *N*(%)	Statistic	*p*-value
Baseline assessment				
Child measures				
Age	11.67 (0.91)	10.95 (0.92)	*t*(107) = 3.95	*p* < 0.001
ELS severity	5.69 (4.50)	5.94 (4.49)	*t*(107) = −0.29	*p* = 0.774
CDI-S total	1.26 (1.22)	2.11 (2.40)	*t*(106) = −2.15	*p* = 0.034
Child race			*X*^2^(5) = 3.54	*p* = 0.618
White	20 (47%)	26 (39%)		
Asian/Asian American	7 (16%)	13 (20%)		
Hispanic/Latin-X	3 (7%)	7 (11%)		
Black/African American	3 (7%)	1 (2%)		
Biracial	7 (16%)	15 (23%)		
Other	3 (7%)	4 (6%)		
Parental income			*X*^2^(3) = 0.60	*p* = 0.897
<$5,000–$35,000	1 (2%)	3 (5%)		
$35,001–$150,000	19 (44%)	31 (47%)		
$150,000+	20 (47%)	27 (41%)		
Don’t know/missing information	3 (7%)	5 (8%)		
COVID-19 assessment				
Age	16.83 (1.22)	15.95 (1.53)	*t*(107) = 3.16	*p* = 0.002
PSS total	15.64 (6.14)	20.24 (6.33)	*t*(106) = −3.73	*p* < 0.001
CES-DC	16.12 (9.24)	23.55 (11.48)	*t*(107) = −3.56	*p* < 0.001

No participant reported having been exposed to someone diagnosed with COVID-19. There were significant sex differences in self-reported impact of COVID-19 based on responses to the CRISIS. Compared to male participants, female participants reported having more COVID-19-related physical symptoms (e.g., cough, shortness of breath, and loss of taste or smell) [*t*(107) = 2.45, *p* = 0.016] and emotional symptoms (worried [*t*(107) = 2.26, *p* = 0.026]; sad [*t*(107) = 3.45, *p* < 0.001]; nervous and anxious [*t*(107) = −2.08, *p* = 0.040]; fatigued [*t*(107) = 3.41, *p* = 0.001]; lonely [*t*(107) = −2.83, *p* = 0.006]; unable to concentrate [*t*(107) = −2.58, *p* = 0.011]; and more negative thoughts [*t*(107) = 2.08, *p* = 0.040]. Finally, consistent with the sex differences on the CES-DC, female participants reported that their mental health was more strongly [*t*(107) = 2.94, *p* = 0.004] and adversely affected by COVID-19 [*t*(107) = 2.57, *p* = 0.012], that the cancellation of events was more difficult [*t*(107) = 3.60, *p* < 0.001], and that they worried more that their food would run out due to a lack of money [*t*(107) = 2.24, *p* = 0.027].

### Survey Completers Versus Non-completers

Adolescents who completed the COVID-19 assessment did not differ from adolescents who did not complete this assessment in age at baseline [*t*(222) = 1.57, *p* = 0.117]; a lower proportion of Black/African American participants and a higher proportion of Asian/Asian American participants completed the COVID-19 assessment than the baseline assessment [*X*^2^(5) = 17.72, *p* = 0.003], but there were no race differences within the group of participants who completed the COVID-19 assessment [*X*^2^(5) = 3.54, *p* = 0.618]. Finally, compared to adolescents who did not complete the COVID-19 assessment, adolescents who completed the COVID-19 assessment had less severe exposure to ELS, lower CDI-S scores, and higher parental income at baseline [*t*(221) = 2.83, *p* = 0.005; *t*(218) = 2.42, *p* = 0.016; *X*^2^(3) = 11.47, *p* = 0.009, respectively].

### Prediction of COVID-19 Perceived Stress and Depressive Symptoms

**FIGURE 3:**
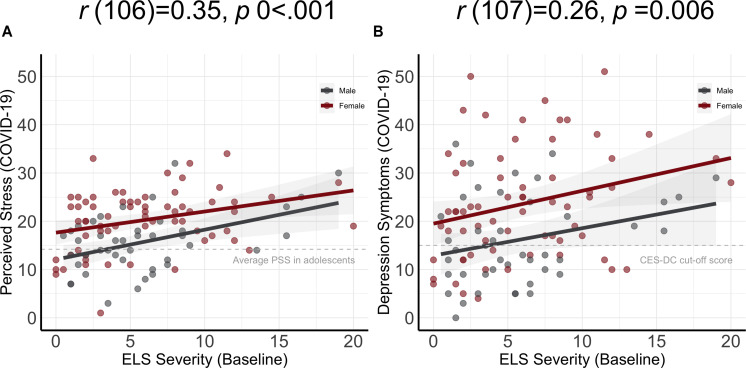
Severity of ELS assessed at baseline is significantly associated with PSS **(A)** and CES-DC **(B)** scores at the COVID-19 assessment. The shaded areas represent 95% confidence intervals of the regression lines.

**FIGURE 4:**
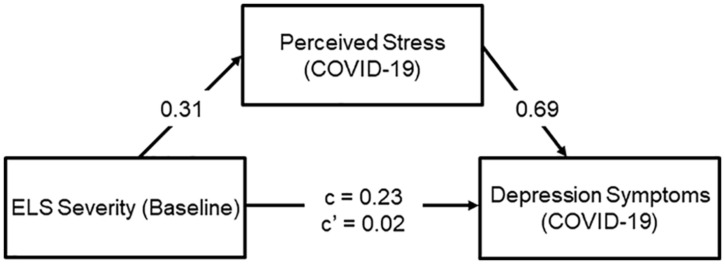
Perceived stress significantly mediates the association between ELS and depression at the COVID-19 assessment. Covariates in this analysis were age at baseline, CDI-S scores at baseline, sex, race, parental income, and age at the COVID-19 assessment. All variables were *z*-scored.

As hypothesized, severity of ELS at baseline predicted severity of both PSS scores [*r*(106) = 0.35, *p* < 0.001] and CES-DC scores *r*(107) = 0.26, *p* = 0.006] at the COVID-19 assessment (see Figure 3). It is also important to note here that rates of stress and depression in this sample at the COVID-19 assessment were high. The mean PSS score in this sample (18.45) is 4.25 points higher than was reported in a validation sample (Cohen et al., 1983), and 63% of our sample scored above the clinical cut-off of 15 on the CES-DC. To examine whether the association between severity of ELS at baseline and depressive symptoms at the COVID-19 assessment was mediated by levels of perceived stress during the pandemic, we conducted a mediation analysis. The mediation model was significant (*R*^2^ = 0.053, *p* < 0.001) and indicated that higher PSS scores at the COVID-19 assessment significantly accounted for the positive association between severity of ELS at baseline and CES-DC scores at the COVID-19 assessment (standardized indirect effect: 0.22; 95% CI: 0.07 to 0.36) Importantly, this mediation of the association between ELS and CES-DC scores by PSS was not moderated by sex (*p* > 0.05; see Figure 4). Importantly, all of these significant findings held when we controlled for the presence of physical symptoms. Finally, as can be seen in Figure 2, the specific COVID-19 stressors that were associated most strongly with perceived stress were those related to changes in restrictions to home confinement [*r*(106) = 0.56, *p* < 0.001], in contact with family [*r*(106) = 0.55, *p* < 0.001], and in social contact [*r*(106) = 0.45, *p* < 0.001]; COVID-related physical symptoms were also associated significantly, but more weakly, with perceived stress [*r*(106) = 0.39, *p* < 0.001].

## Discussion

Early life stress (ELS) is a well-documented precipitant of depression later in life (LeMoult et al., 2019b). In particular, stress experienced during early development such as childhood (Teicher et al., 2016) may cause sustained neurobiological changes that increase sensitivity to future stressors (Post, 1992). In this context, the ongoing COVID-19 pandemic is likely to cause widespread difficulties in mental health (Holmes et al., 2020), including depression, particularly among adolescents who are at elevated risk for depression due to having experienced ELS. In the present study, we tested this formulation by leveraging a richly characterized sample of adolescents whom we had been following for several years. We obtained data on levels of depressive symptoms, stress, and contextual factors related to mental health functioning during the pandemic in Northern California, an early epicenter of the pandemic and one of the first areas in the United States to implement isolation directives. Importantly, these adolescents had very low levels of depressive symptoms at the time of recruitment and had been carefully phenotyped with respect to previous exposure to ELS. We found here that greater exposure to ELS predicted higher levels of symptoms of depression during the pandemic. Further, higher levels of perceived stress during the pandemic significantly mediated this association, even after controlling for age, sex, race, income, and earlier levels of depression. Our results highlight the importance of considering early adverse experiences when evaluating mental health risk for adolescents and suggest that psychosocial interventions that reduce the perception of stress and increase social connectedness will mitigate this risk.

Our findings are consistent with early reports from the Hubei province in China, which documented increased levels of depression and anxiety in adolescents during the COVID-19 pandemic (Xie et al., 2020). In addition, emerging research in the United States points to a worsening of mental health in youth with pre-existing mental illness following school closures during the pandemic (Lee, 2020). A key strength of our study, however, is that we *prospectively* predicted mental health functioning during COVID-19 using data we had acquired 3–7 years prior to the pandemic in a sample of adolescents who, at that time, had minimal symptoms of depression. Notably, the adolescents in our full sample who participated in the current COVID-19 study had *lower* levels of depression at baseline than did the adolescents who did not participate and, yet, over 60% of the sample endorsed current levels of depression that warrant additional screening. Thus, our results are likely to be underestimating the adverse impact of ELS on depression risk in adolescents. Moreover, in addition to identifying ELS and female gender as significant risk factors for the development of depression in adolescents during the COVID-19 pandemic, we drew on Post’s (2007) formulation of kindling to identify and test a mechanism—perceived stress—that mediates this association and, importantly, that clinicians can target. Finally, we also found that the daily life changes in response to quarantine and isolation directives adolescents reported as being most stressful primarily concerned social stressors. Thus, our study contributes important new findings that can inform clinical practice in identifying and treating at-risk youth and guide public health policies seeking to reduce viral transmission while considering the mental health implications of such directives.

Previous studies have linked higher rates of depression to large-scale crises that affect public and economic health – including earthquakes (Tang et al., 2015), wildfires (Zeller et al., 2015), and terrorist attacks (Liu et al., 2020). The situation generated by the COVID-19 pandemic, however, is unprecedented in terms of its consequences of mandated social isolation (Brooks et al., 2020; Golberstein et al., 2020). The mental health consequences of stressors resulting from extreme social upheaval are almost certain to be compounded by living situation instability, food insecurity, and other financial hardships that many are experiencing during the pandemic. Nevertheless, unlike economic instability, perceived stress is a modifiable target for accessible interventions that may prevent the exacerbation of depressive symptoms during a time when access to standard care and social support is dramatically reduced. For example, cognitive reframing can be implemented *via* telemedicine. Tele-mental health has been shown to be as effective as in-person therapy, including for adolescents and individuals with mood disorders (Slone et al., 2012; Arnberg et al., 2014); such therapies may help adolescents and parents cope with stressors and find meaning in response to a collective trauma, fostering resilience and social connectedness.

We should note limitations of our study. We assessed adolescents during the initial weeks of the Bay Area quarantine and social distancing policies enacted in response to COVID-19; given shelter-in-place directives, however, our data are necessarily based on responses to online self-report measures spanning the time period when quarantine began. In this context, our measures of stress and depressive symptoms during COVID-19 were assessed concurrently. It will be important that future studies examine longer-term effects on adolescents of pandemic-related social restrictions (Andrews et al., 2020). In addition, because depression is one of the most prevalent mental health difficulties in adolescence, we focused in this study on depressive symptoms as a clinical outcome; additional studies are needed to test whether our findings generalize to other mental health conditions (e.g., bipolar disorder, psychosis, and suicidality). Further, only about half of the original participants completed the COVID-19 assessment; importantly, however, completers had less severe exposure to ELS, lower baseline depressive symptom scores, and higher parental income at baseline, making the relatively high levels of stress and depressive symptoms at the COVID-19 assessment all the more striking. The San Francisco Bay Area was one of the first areas to implement widespread and stringent quarantine and social distancing policies; it will be important that researchers assess adolescents in other geographic areas in which similar policies were implemented later and examine whether the duration of social isolation is associated with adverse mental health outcomes. Finally, if we are to foster mental health in a time of a globally stressful event, future studies must also identify sources of resilience that can buffer the onset of depressive symptoms, particularly in high-risk youth.

This study is the first to assess longitudinally the relation between ELS and depression during the COVID-19 pandemic. Our findings indicate that it is critical that adolescents, especially females, who have experienced ELS be monitored regularly for safety and mental health. Certainly, it is too early to determine the long-term impact of the COVID-19 pandemic on mental health. Nevertheless, our study, as well as those from other investigators around the world, shows that high levels of depressive symptoms are likely to occur during the pandemic. We also know that many families will endure financial hardship and an absence of social support even after the quarantine and social isolation policies have been lifted. Thus, our results highlight the importance of implementing accessible psychosocial interventions designed to reduce stress perception and increase social connectedness during this difficult time.

## Data Availability Statement

The datasets presented in this study can be found in online repositories. The names of the repository/repositories and accession number(s) can be found below: https://github.com/lrborchers/els_covid.git and https://rpubs.com/lrborchers/707001.

## Ethics Statement

This study was approved by the Institutional Review Board at Stanford University. Participants provided written informed assent and their parent(s)/legal guardian(s) provided written informed consent.

## Author Contributions

All authors contributed to the design and conduct of the study and to the writing of the manuscript.

## Conflict of Interest

The authors declare that the research was conducted in the absence of any commercial or financial relationships that could be construed as a potential conflict of interest.

## Footnotes

1 https://github.com/nimh-mbdu/CRISIS
